# Chromotropism of Iron(II) Complexes with Non-Symmetric Heterocyclic Ligands: Polarity Sensing in Aqueous Urea Solutions

**DOI:** 10.3390/nano15080598

**Published:** 2025-04-13

**Authors:** Raffaello Papadakis

**Affiliations:** Department of Forest Biomaterials and Technology, Swedish University of Agricultural Sciences, 756 51 Uppsala, Sweden; rafail.papadakis@slu.se

**Keywords:** chromotropism, 4,4′-bipyridine, iron(II) complexes, solvatochromism, urea sensing, specific solvent–solute interactions

## Abstract

This study reports the synthesis and characterization of two solvatochromic pentacyanoferrate(II) complexes. Their structural design incorporates ligands with flexible xylylene bridges and distinct heterocycles—one combining 4-dimethylaminopyridine (DMAP) with 4,4′-bipyridine, and the other isoquinoline with 4,4′-bipyridine. Their structural diversity enables the complexes to engage in a broad range of solvent–solute interactions, providing valuable insights into the behavior of solvents and media with regard to polarity, through sizable solvatochromic shifts. Their solvatochromism is examined using a set of nine solvents and solvent mixtures. The solvatochromic sensitivities to polarity changes, expressed through a variety of polarity parameters and functions, are determined. Moreover, using a set of four complementary linear solvation energy relationships (LSERs), the roles of solvent polarity, solute–solvent interactions and the molecular responsiveness of the two compounds to different media are investigated. Additionally, their dipole moments in the ground and MLCT-excited states are determined using a suitable model, namely that of Suppan and Tsiamis. As a step further, the polarity sensing aptitude of the two solvatochromic compounds is examined in aqueous urea solutions at varying urea concentrations. The solvatochromic sensitivity of the two compounds is compared with that of a model cyanoferrate(II) complex, Fe^II^(CN)_2_(phen)_2_. The two compounds clearly surpass the sensitivity of Fe^II^(CN)_2_(phen)_2_ with subtle solvent polarity changes induced by varying the urea concentration. An LSER describing and predicting the solvatochromic effects in aqueous urea is developed and tested.

## 1. Introduction

Cyanoferrate(II) complexes have gained significant attention in recent years due to their versatile applications in various fields, including catalysis [1,2,3], materials science,[4,5,6], and medicinal chemistry [7]. These complexes are particularly renowned for their unique electronic properties and their ability to form stable complexes with a variety of ligands [8,9,10]. Recent trends in the study of cyanoferrate(II) complexes have focused on enhancing their functional properties through innovative ligand design and exploring their potential in new applications [11]. One of the key features in developing these complexes is the incorporation of 4,4′-bipyridine as a ligand [12]. 4,4′-Bipyridine is a bidentate ligand that can coordinate with metal centers to form robust, versatile structures. Its ability to undergo redox reactions and its electrochromic properties make it an attractive component in the design of multifunctional materials [12]. The involvement of 4,4′-bipyridine in cyanoferrate(II) complexes has led to the development of compounds with enhanced stability, improved electronic properties, and increased responsiveness to environmental changes [13,14,15,16,17,18,19,20].

Medium responsive materials, which change their properties in response to external stimuli such as temperature, pH, or solvent polarity, constitute an exciting area of research of high scientific and technological relevance [21,22,23]. Pentacyanoferrate(II) complexes with 4,4′-bipyridine ligands have shown promising results as medium responsive materials [12]. These materials can exhibit significant changes in their optical and electronic properties when exposed to different solvents or solvent mixtures. This solvatochromic behavior is beneficial for sensing applications, where the material’s response to environmental changes can be used to detect and measure various parameters/properties of the environment. The combination of cyanometallate complexes with 4,4′-bipyridine and related ligands and their medium responsive properties opens up new possibilities for the design of advanced materials [12,24,25]. These materials can be tailored for specific applications, such as sensors [26] and smart coatings [27], where their ability to respond to environmental changes can be harnessed for practical use. The ongoing research in this field aims to further understand the mechanisms behind these responses and to develop new materials with enhanced performance and broader applicability. For these reasons, various attempts have been made to investigate solvation effects in diverse media, with a special interest in solutions (aqueous or not) of molecules acting as modifiers [18,28,29]. However, studies on the alteration of the ionicity of solute indicators due to the dielectric effects of the medium remain scarce. Embracing these facts, this work features two pentacyanoferrate(II) complexes that are synthesized and fully characterized, exhibiting intense solvatochromic properties. Their solvatochromism is examined in various solvents and solvent mixtures. These complexes feature ligands with flexible xylylene bridges and distinct heterocycles: one bearing DMAP and 4,4′-bipyridine, and the other isoquinoline and 4,4′-bipyridine. This structural diversity leads to a remarkable solvatochromic behavior, enabling a variety of intermolecular interactions between solvents and complexes acting as solutes. Linear solvation energy relationships are employed to investigate their dependencies on solvent polarity and their responsiveness to different media. A further goal of this work is to explore polarity sensing and solvation effects in aqueous urea. There are several reasons why was chosen as a key polarity modifier in water. Firstly, urea is widely used in numerous processes and materials of industrial importance, most prominently in urea–formaldehyde [30,31] and urea–phenol–formaldehyde resins [32], which have immense applications in adhesives [33,34,35], and coatings of global technological significance [36,37]. Additionally, urea is considered a bio-based compound (depending on the production process), and its use in new “green” adhesive formulations and polymers is currently being highly promoted [38]. To facilitate its use in novel applications, its properties as a solute require more investigation. Furthermore, the study of aqueous urea solutions is crucial for understanding their dielectric properties and the specific effects of H-bonding, specific solute–solvent interactions. This study also aims to investigate the solvent–solute interactions in the presence of urea in water solutions of the title solvatochromic compounds/probes. FTIR and UV-Vis spectrophotometric experimental results and different mathematical models are employed to rationalize the complex solvation effects in aqueous urea solutions.

## 2. Results and Discussion

### 2.1. Synthesis and Characterization

**Scheme 1:**
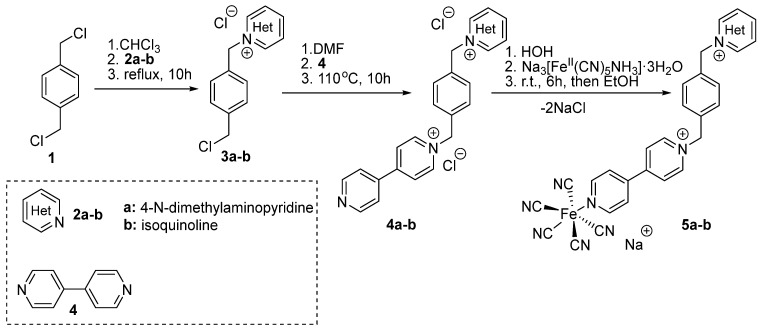
Synthetic route to solvatochromic products **5a-b**.

The solvatochromic pentacyanoferrate(II) complex **5a** was synthesized and characterized previously by [18]. An analog of **5a**, lacking the polar dimethylamino functional group and further benzanullated (complex **5b** involves an isoquinoline-bearing ligand, see Scheme 1) was synthesized for the first time.

Two different heterocyclic compounds were used for the synthesis of the ligands (**4a-b**). 4-Dimethylaminopyridine (**2a**) and isoquinoline (**2b**) reacted in a 1:2 ratio with α,α’-dichloro-*p*-xylene (**1**), while 4,4′-dipyridine (**4**) reacted in a 2:1 ratio to yield the respective products (**4a-b**). Through the reaction of products **3a** and **3b** with 4,4′-dipyridine, products **4a-b**, which were subsequently used as “ligands”, were produced. The general synthesis method of the heterocyclic products **4a-b**, as well as their structural evaluation, is described in Section 4. Importantly, excess electrophile was used to avoid di-substitution, as the introduction of the first heterocyclic group activates the other electrophilic carbon of α,α′-dichloro-*p*-xylene. The resulting 4,4′-dipyridine-bearing compounds were further used as ligands in pentacaynoferrate(II) complexes **5a-b**. The synthesis of the latter Fe(II) complexes was achieved via the reaction of ligands **4a-b** with Na_3_[Fe^II^(CN)_5_NH_3_]·3H_2_O], a reagent bearing NH_3_ as an easily displaced compound.

Both solvatochromic complexes **5a** and **5b** were characterized using various analytical methods, including NMR (^1^H and ^13^C) and FTIR spectroscopy, as well as thermogravimetric (TG) and elemental analyses. The characterization and synthesis of compound **5a** was previously reported by [18]. Through combined TGA and elemental analyses, the isolated and purified compound **5b** was determined to correspond to a hexahydrate, with the molecular formula C_32_H_23_FeNaN_8_·6H_2_O (**5b**·6H_2_O). (For further details, see the Section 4).

### 2.2. Solvatochromism

**Scheme 2:**
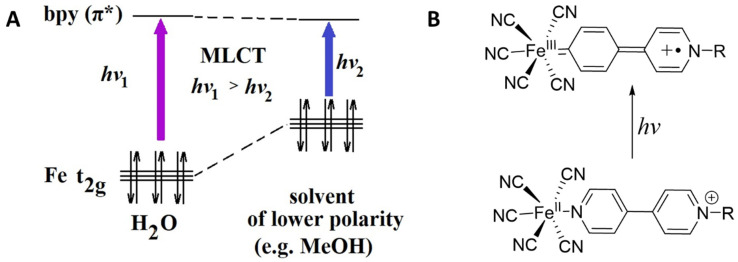
(**A**) Illustration of the ${Fe}^{II}(dp)\to \pi^{*}(bpy)$ transition observed in complexes **5a-b** and comparison of the energy gaps in two solvents of different polarity. (**B**) Scheme depicting the “solvatochromic backbone” of compounds **5ab** and the photoinduced MLCT transition and how the structure is influenced.

Compounds **5a** and **5b** exhibit marked solvatochromism, similar to other ferrocyanide(II) complexes encompassing 4,4′-dipyridine-based ligands (see recent works by Papadakis and coworkers) [13,15,17,18,20]. It is worth nothing that compounds **5a** and **5b** ligand’s have a flexible backbone, which can potentially diversify solvent–solute interactions and give rise to unique solvation phenomena. Recently, the unique structural properties of **5a** have been tested in the context of glucose sensing [18]. In the report, it shows that there is a bimodal chromotropic behavior: with one mode encountered in molecular solvents and their mixtures, and another mode in glucose solution associated with the attenuation of ionicity of **5a** in aqueous glucose [18]. The solvatochromism of ferrocyanide(II) complexes encompassing 4,4′-dipyridine-based ligands is associated with the medium influenced character of the ${Fe}^{II}(dp)\to \pi^{*}(bpy)$ transition (see Scheme 2A). As shown in Scheme 2A, the energy gap between the ground and MLCT excited state of **5ab** is larger in highly polar solvents such as water than in less polar solvents, e.g., MeOH. This effect drastically influences the color of these compounds by inducing sizable bathochromic shifts in the visible spectra upon decreasing solvent polarity. The “solvatochromic backbone” experiencing this effect involves the 4,4′-bipyridinium part of the molecule and the pentacyanoiron(II) part of the complex (see Scheme 2B).

Here, it is attempted to go a step further and investigate the properties of relative compound **5a**, which differs only in the use of an N-heterocycle opposite to 4,4′-dipyridine, specifically isoquinoline instead of DMAP (the latter appearing in the structure of **5a**). Due to the lower dipolarity of isoquinoline compared to DMAP and the lack of hydrogen bonding possibilities, the chromotropic behavior of **5b** is anticipated to be influenced mostly by the dipolarity and likely by the polarizability of the medium. This can lead to a polarity indicator that encompasses only one hydrogen bond sensing side, that of the -Fe^II^(CN)_5_ moiety which plays a dominant role in the observed solvatochromism, and thus, this structural feature could attenuate the role of other sites of the molecule which give rise to solute–solvent interactions of lower importance for the core transition, e.g., the H-bonding of the type Sol-H^…^NMe_2_py (where Sol is a solvent with H-Bond donating aptitude and NMe_2_py being DMAP)]. To have a meaningful comparison of the two complexes the same molecular media were used for **5a** as for **5b**. More details about the used solvents and solvent mixtures are found in Table 1.

**Figure 1:**
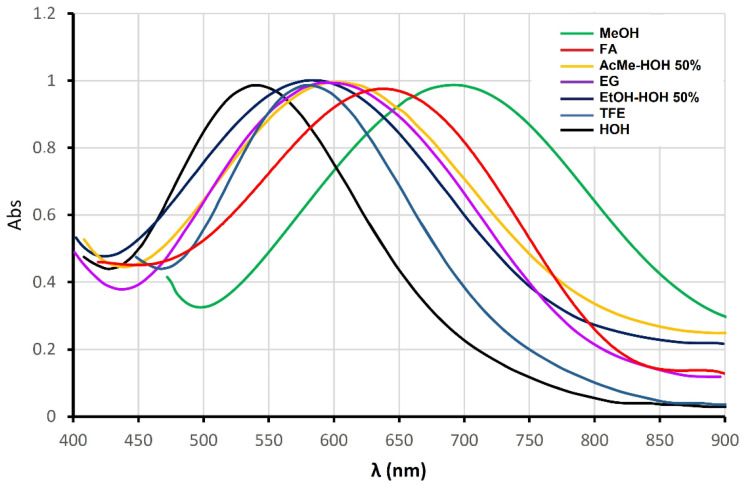
Normalized visible absorption spectra of compound **5b** (MLCT band) in different solvents.

Through the spectrophotometric study of compounds **5a** and **5b** (results in Table 2 and Figure 1) in various solvents and solvent mixtures, intriguing effects were observed. Qualitatively, the most noticeable observation is the negative solvatochromism of both compounds, corresponding to a bathochromic shift in the MCLT band as the solvent polarity decreases. Quantitatively, compound **5a** exhibited a maximal bathochromic shift of $\Delta_{HOH}^{NMF}\lambda_{CT}=92\;\;nm$ (or $\Delta_{HOH}^{NMF}{\tilde{v}}_{CT}=-2766{\;cm}^{-1}$) between water (the solvent with the highest *E_T_*(30) value in this study) and NMF (the solvent with the lowest value of Reichardt’s polarity scale (*E_T_*(30)), the basis of which is solvatochromic model compound **6**). For compound **5b**, the corresponding shift was 88 nm (or −2606 cm^−1^). Noteworthy, the corresponding difference in *E_T_*(30) scale between water and NMF is $\Delta_{HOH}^{NMF}\lambda_{CT}=75.4$ nm, i.e., lower than the corresponding values of either of **5a** and **5b**. This finding implies a higher sensitivity of **5a** and **5b** compared to model compound **6** between these polar solvents. Interestingly, while NMF has the lowest *E_T_*(30) value in this study, it was not the solvent in which the highest solvatochromic shift was observed. Instead, MeOH exhibited the most significant shift, with $\Delta_{HOH}^{MeOH}\lambda_{CT}=137\;nm$ $(or\;\Delta_{HOH}^{MeOH}{\tilde{v}}_{CT}=-3841{\;cm}^{-1}$) for **5a** and 153 nm (−4118 cm^−1^) for **5b**. This is not surprising, as NMF, despite its low *E_T_*(30) value due to its low Lewis acidity (significantly lower than MeOH), also has one of the highest dielectric constants (ϵ = 182.4), indicating stronger dipolarity among molecular solvents (including water). Indeed, the author has recently reported on an observation of the high polarity-driven behavior of NMF in binary solvent mixtures [13]. This finding suggests that both compounds are more influenced by dielectric effects than by the Lewis acidity of the medium. Furthermore, it underscores the importance of interpreting solvatochromic shifts using various scales and parameters (empirical or otherwise) to gain a better understanding of the dominant solute–solvent interactions.

Moreover, small but noticeable differences in the measured MLCT maxima wavelengths were observed when comparing **5a** to **5b** in different solvents. The most prominent differences are a 19 nm shift observed in neat MeOH and a 10 nm shift in FA, with the band of **5b** being more redshifted than that of **5a** in both cases. Additionally, the general observation in most solvents/mixtures examined, excluding NMF and EG, is that the visible MLCT band of **5b** is either centered at an identical wavelength or redshifted compared to **5a**. Although it is difficult to draw definitive conclusions from these observations, the DMAP group is likely capable of pushing electron density into the “solvatochromic backbone” of the molecule (i.e., 4,4′-bipyridine-Fe^II^(CN)_5_) more effectively than isoquinoline. It has been shown earlier by Papadakis and Tsolomitis [20] that electron-withdrawing substituents bound to the N-end of the 4,4′-bipyridine-Fe^II^(CN)_5_ backbone result in redshifts in the visible spectra of compounds relative to **5a** and **5b**. For a thorough study focusing on these effects, please refer to the relevant literature [20].

### 2.3. Types of Solute–Solvent Interactions

Compounds **5a** and **5b** can interact with solvent molecules in various ways due to the diversity of their functional groups. Understanding these interactions is crucial for rationalizing solvation phenomena. This study identifies the following main types of solute–solvent interactions.

**Scheme 3:**
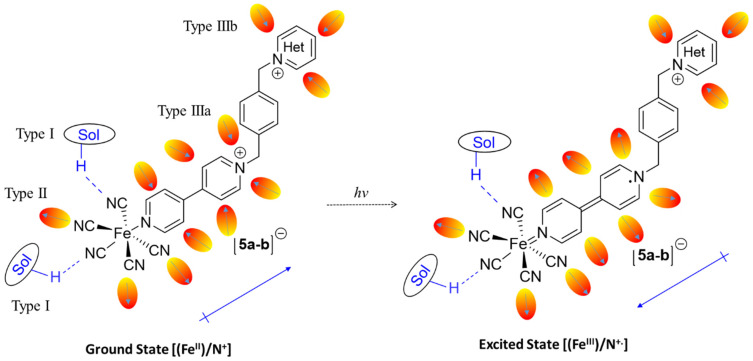
Illustrations representing the interactions of compounds **5a** and **5b** with solvent molecules (dipoles) in the ground and excited state after reorientation (Sol: corresponds to solvent).

The (C≡N)^−^ groups of the pentacyanoferrate(II) “head” of the molecules can effectively hydrogen bond to a variety of hydrogen bond donor (HBD) molecules, such as hydroxylated solvents (e.g., water, alcohols) and amides (e.g., FA and NMF) [13,20]. This type of interaction is identified here as **Type I**, see Scheme 3. These interactions are highly relevant to the chromic behavior of pentacyanoferrates(II) since H-bonding attenuates the Fe^II^-CN bond strength. Hence, MLCT becomes less efficient, and its energy increases. This has a direct impact on solvatochromism. Indeed Brønsted acids capable of protonating the CN groups while remaining bound to the Fe(II) center, have been found to drastically change the chromic and non-linear optical (NLO) properties of pentacyanoferrate(II) complexes [42].

Moreover, the (C≡N)^−^ groups can also interact specifically with solvent dipoles (anion-dipole interactions, **Type II** interactions, Scheme 3). Such interactions clearly influence the MLCT energy and therefore the solvatochromism of **5a** and **5b** in a similar fashion as in **Type I** interactions, i.e., by energetically disfavoring the MLCT [20].

The charged backbone of the ligand, i.e., the bipyridinium part adjacent to the pentacyanoferrate(II), also exhibits an aptitude to interact with the dipoles of solvent molecules through cation–dipole interactions (see Scheme 3). Support for this type of interaction was provided by ^1^H-NMR studies on pyridinium salts [43]. These interactions (here called **Type IIIa**) can influence the solvatochromism of **5a** and **5b** by attenuating the electron-withdrawing aptitude of the bipyridinium positively charged core. Such interactions will obviously hamper the transfer of charge from iron(II) to the heterocycle (note: the MLCT transition is the ${Fe}^{II}(dp)\to \pi^{*}(bpy)$ transition; *vide supra*).

On the other hand, bipyridinium as well as the quaternized N-heterocycle (DMAPyridinium for **5a** and quinolinium for **5b**) are capable of developing cation–dipole interactions with dipoles of polar solvents. (**Type IIIb** interactions). In contrast to quinoline, DMAP can also develop hydrogen bonding with HBD-solvents such as water, formamide, and methanol (MeOH) as a result of the mild HBA capacity of the dimethylamino group of DMAP (**Type IV**).

The latter two types (**Type IIIb** and **IV**) of interactions do not directly affect the core of the molecule and hence, their contribution to the solvatochromic is rather minor. Nonetheless, as described above, small shifts in the visible spectra of **5a** and **5b** can be attributed to the heterocyclic substituent and explained based on **Type IIIb** and **IV** interactions, which are dependent on the nature of the side-heterocycle.

### 2.4. Using LSERs to Rationalize the Solvatochromic Effects

To elucidate the solute–solvent interactions that influence the solvatochromism of **5a** and **5b**, selected LSERs were employed (see the Section 4). The choice of LSERs was based on a series of previous studies on pentacyanoferrate(II) complexes with variously substituted 4,4′-bipyridines as ligands [13,14,17,18,19,20].

Correlations between measured ${\tilde{v}}_{CT}$ values for **5a** and **5b** using the LSER models (Equations (1)–(4)) help in understanding the solvent polarity parameters that influence the observed solvatochromism of both compounds. The correlation between ${\tilde{v}}_{CT}$ and $E_{T}^{N}$ resulted in a good fit (*r*^2^ = 0.712 for **5a** and *r*^2^ = 0.617 for **5b**; see Table 3 for more details) with a positive slope of 10.54 × 10^3^ cm^−1^ for **5a** and 10.39 × 10^3^ cm^−1^ for **5b** (see Table 3). $E_{T}^{N}$ is a polarity scale encompassing Lewis acidity and dipolarity/polarizability character [39] and these correlations are in line with the specific role of Lewis acidity of solvents in **Type I** interactions as well as with the role of dipolarity on **Type II** and **Type III** interactions and their overall influence on solvatochromism. Similar results have been shown by a range of recent studies and are characterizations of ferrocyanides [14,18,19,20]. The positive slopes in both cases indicate a negative solvatochromic effect, corresponding to bathochromism (lowering of ${\tilde{v}}_{CT}$) when solvent polarity increases.

**Figure 2:**
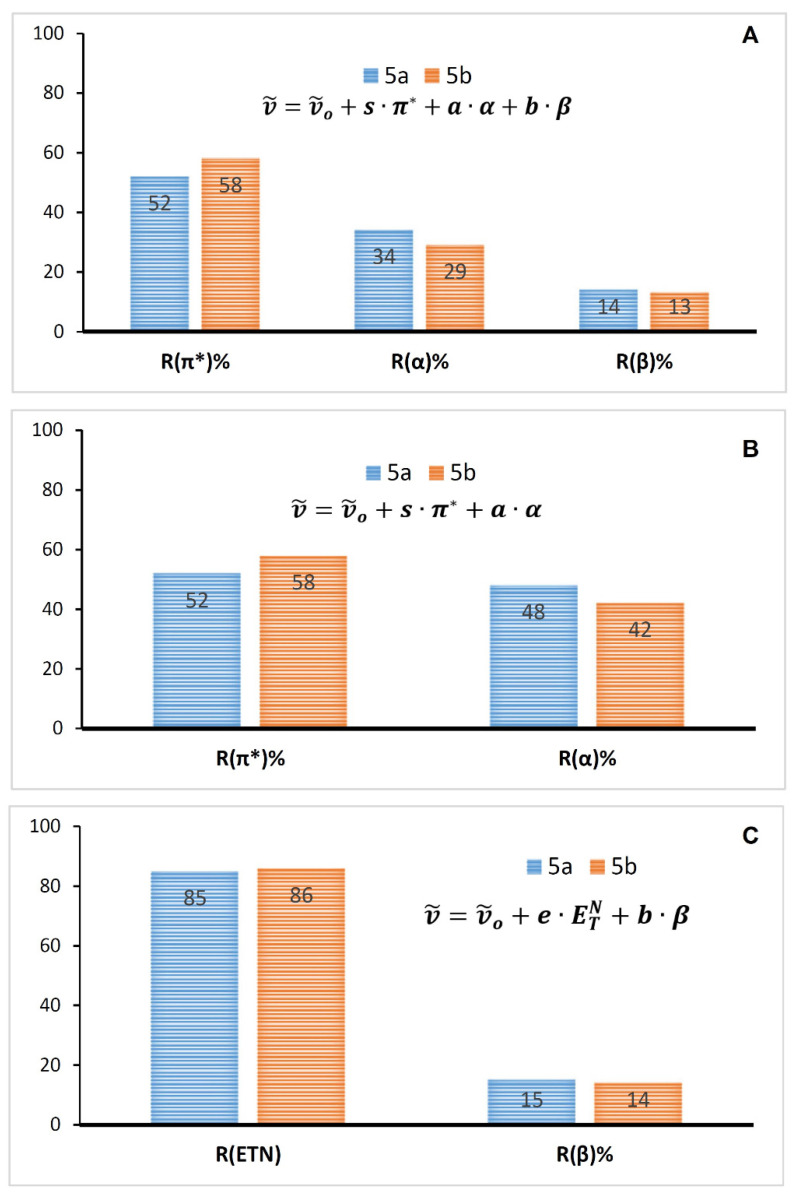
Bar graphs depicting the relative importance of the polarity parameters involved in Equations (2) (**A**), (3) (**B**), and (4) (**C**).

Due to the fact that Lewis acidity and dipolarity/polarizability are entangled in the empirical scale of Reichardt, $E_{T}^{N}$, Kamlet–Taft–Abboud (KAT) equation in its triparametric form (see Section 4; Equation (2)) can help to separate acidity (specifically HBD acidity), basicity (HBA basicity), and dipolarity/polarizability of solvents [44]. The triparametric linear fits of the spectrally obtained ${\tilde{v}}_{CT}$ values to the three parameters π*, *α*, and *β* for **5a** and **5b** were satisfactory (*r^2^* = 0.881 for **5a** and 0.847 for **5b**; see Table 3 for more details). A general observation for both **5a** and **5b** is that positive coefficients (also known as sensitivities) were obtained for π* and *α,* whereas negative ones were obtained for *β* (see Table 3). The positive coefficients *s* (corresponding to π*) are in line with the assumption of negative solvatochromism since the increase in dipolarity/polarizability triggers a hypsochromic effect in the vis-spectra of **5a** and **5b** (characteristic of most bipyridine-bearing pentacyanoferrate(II) complexes) [20]. The positive coefficient of *α* parameter (*a*) is also in line with the expected effect of HBD acidity on **5a** and **5b** (see **Type I** interactions above). On the other hand, the negative response to changes in parameter *β* (reflected in the negative *b* values for both compounds, see Table 3) is potentially associated with the channeling of electron density towards the core of the complex induced by interactions with solvent molecules acting as HBA/Lewis bases. Such an effect is expected to have an opposite impact compared to acidity or dipolarity, as it can enhance the electron-pushing towards the electron-deficient parts of compounds **5a** and **5b**. Additionally, solvents interacting with the pyridinium entities of **5a** and **5b** in a **Type IIIa** fashion (*vide supra*)—for example, by interacting with the *ortho*-hydrogen atoms of the bipyridinium—could lead to further polarization, resulting in a more energetically favored MLCT when basicity increases, inducing bathochromism while solvent basicity increases. Such phenomena have not been extensively studied, yet there are indications of the effects of HBA and Lewis basicity on the solvatochromism of pentacyanoferrate(II) complexes [19]. Nonetheless, the overall role of basicity on the studied solvatochromic effects was found to be statistically much less important than the role of dipolarity/polarizability and HBD/Lewis acidity ($R_{\beta }$ was determined to be 14% for **5a** and 13% for **5b**; see Table 4 and Figure 2A). For that reason, a report has recently attempted to employ a reduced form of the KAT equation having two parameters instead of three [19]. This approach was also attempted in this study. Specifically, parameter *β* is neglected in the analysis of solvatochromism of **5a** and **5b**. In this study, the use of Equation (3) (involving merely π*, *α*; see methods section for details) resulted in very good fitting, being comparable to the triparametric KAT equation. Through statistical analysis, it was found that parameter π* exhibits higher importance than parameter *α* and this effect is slightly more pronounced in compound **5b** than **5a** (see Table 4 and Figure 2B). This finding is in line with the structural diversity of **5a** and **5b** and specifically the difference in side-pyridinium substituents; DMAP for **5a** and quinolinium for **5b**. Because isoquinoline compared to DMAP lacks hydrogen bonding capacity, the chromotropic behavior of **5b** is more significantly affected by the dipolarity and polarizability of the medium. Interestingly, the application of Equation (3) can be viewed as a disentanglement of Equation (1). While the latter (Equation (1)) fits the spectrally obtained ${\tilde{v}}_{CT}$ to $E_{T}^{N}$, Equation (3) breaks down the fitting into the two components of $E_{T}^{N}$, namely Lewis acidity and dipolarity/polarizability, which are described in Equation (3) through the linear combination of π* and *α*. Indeed, the connection of *E_T_*(30) to parameters π* and *α* has been previously reported (e.g., *E_T_*(30) = 31.2 + 15.2·*α* + 11.5·π*, *r*^2^ = 0.9585) [45].

Finally, an LSER complementary to the KAT equation was further used (see Equation (4)). The latter involves $E_{T}^{N}$ expressing a combination of dipolarity/polarizability and Lewis acidity with parameter *β* expressing HBA basicity. This LSER can be regarded as analogous to the Krygowski–Fawcett equation [46]. In this form, Equation (4) provides the benefit of using KAT parameter *β* which allows to draw a complementary conclusion to that of the KAT equation (Equation (2)). Interestingly, Equation (4) provides qualitatively the same results as KAT equation. While positive sensitivities to $E_{T}^{N}$ (10.14 × 10^3^ cm^−1^ for **5a** and 10.06 × 10^3^ cm^−1^ for **5b**) were obtained, negative sensitivities to the KAT parameter *β* were observed (−0.288 × 10^3^ cm^−1^ for **5a** and −0.238 for **5b**). The interpretation of this finding is the same as in the KAT equation (*vide supra*). Additionally, the sensitivities to $E_{T}^{N}$ were very close to those obtained through Equation (1) (involving solely Reichardt’s polarity scale, see Table 3 for details). This underlines the minimal role of parameter *β* which is manifested through the very low relative importance of this parameter (15% and 14% for **5a** and **5b**, respectively; see Table 4 and Figure 2C). The complementarity of Equations (2) and (4) is also manifested through the very close relative importance of parameters $E_{T}^{N}$ and π* and *α* the latter two combined i.e., ${\%R}_{E_{T}^{N}}=85\%\;≅\left(\%R_{\pi *}+{\%R}_{\alpha }\right)=\left(52\%+34\%\right)=86\%\;$ for **5a** and ${\%R}_{E_{T}^{N}}=86\%\;≅\left(\%R_{\pi *}+{\%R}_{\alpha }\right)=\left(58\%+29\%\right)=87\%\;$ for **5b**. The overall interpretation of the qualitative and quantitative results obtained through Equations (1)–(4) indicates that the dipolarity/polarizability of the medium, along with HBD and/or Lewis acidity, are the most important solvent polarity features for the observed solvatochromic effects, in both compounds examined. The results clearly indicate a negative solvatochromic effect, and the impact of HBA basicity is found to be of minor importance, given the set of solvent/solvent mixtures examined, which, interestingly, involves solvents of relatively high basicity (e.g., NMF with *β = 0.80*, glycerol with *β =* 0.87, and MeOH with *β* = 0.66).

### 2.5. Determination of Electronic Ground and Excited State Dipole Moments

Determining the dipole moments of solvatochromic dyes is essential for understanding their molecular polarity and optimizing their electronic properties. Knowledge of dipole moments in the ground and excited states can assist in enhancing the performance of these dyes in applications like sensors [18,47] and molecular electronics [48], where the precise tuning of electronic properties is crucial [49]. Additionally, it helps predict spectroscopic behavior, providing valuable insights into the electronic structure and bonding patterns of these dyes. Taking these facts into account, ground and excited state dipole moments of **5a** and **5b** were determined. The dipole moments were determined by employing a model introduced by Suppan and Tsiamis [50] (see Section 4, Equations (5) and (6)) which allows for the determination of ground and excited dipole moments of non-emissive solvatochromic compounds [51]. A report recently determined the dipole moment difference between the ground and MLCT-excited state of **5a**; nonetheless [8], the current study included an additional molecular solvent (TFE) which was taken into account and for a complete comparison with the results obtained for **5b** (also including TFE). The complete results of the application of the model for **5a** and **5b** are presented in Table 5. The obtained values for **5a** were very close to the reported ones in a recent publication by the authors. Small differences in dipole moments between **5a** and **5b** were observed and **5b** was found to exhibit a slightly more polar excited state than **5a** as well as a larger difference $\vec{\mu_{e}}-\vec{\mu_{g}}$ which is in line with the overall more dipolar nature of **5b** compared to **5a** (see discussion above) with $\vec{\mu_{e}}=-1.423\vec{\mu_{g}}$ for **5a** and $\vec{\mu_{e}}=-1.479\vec{\mu_{g}}$ for **5b**. Qualitatively, the model predicts a higher dipolar moment for the MLCT excited state compared to the ground state in both cases of compounds which is in line with the hypothesis of negative-solvatochromism. Practically, an increase in “pure” dipolarity expressed by an increase in the function: $\varphi \left(\epsilon ,n^{2}\right)=\varphi \left(\epsilon \right)-\varphi \left(n^{2}\right)$ which excludes polarizability ($\varphi \left(n^{2}\right))$ of the medium results in an increase of $E_{CT}$ i.e., it induces hypsochromism.

### 2.6. Polarity Sensing in Aqueous Urea

The intense solvatochromic behavior of compounds **5a** and **5b** can be conveniently used to quantify solvent polarity in aqueous solutions of polarity modifiers. The sensing aptitude of compound **5a** in aqueous glucose solutions has been previously reported [18]. In this study, we attempt to quantify and rationalize the behavior of the polarity sensors **5a** and **5b** in aqueous urea solutions. To the best of our knowledge, studies on solvation effects in aqueous urea solutions are scarce and mainly focus on their dielectric properties [52]. Nonetheless, there are only a few studies pertaining to the use of solvent polarity indicators that could probe specific effects such as H-bonding solute–solvent interactions [19]. To shed more light on this, the influence of urea concentration on polarity in water-based solutions is investigated using compounds **5a** and **5b**.

***Background studies:*** In the study of urea–water solutions, it has been observed that dimers of urea form up to a concentration of 2 M, while above this concentration, urea aggregates into chain-like structures [53]. Rezus and Bakker, using polarization-resolved mid-infrared pump–probe spectroscopy, measured the orientational dynamics of water molecules and examined the rigidity of hydrogen bonds in urea–water mixtures. They found that water structured networks are retained in the mixture, and urea interacts with only a few water molecules, leading to the formation of specific urea–water molecular arrangements [54]. Moreover, Soper, Castner, and Luzar reported that urea can form hydrogen bonds with either water or urea molecules without significant preference [55]. Additionally, quantitative analysis of spectra by Hayashi et al. showed that the number of hydration water molecules is approximately two per urea molecule for concentrations below 5.0 M, while previous molecular dynamics studies predicted approximately six water molecules [52]. Different authors have shown that there is a nearly linear increase in static permittivity with increasing urea content (see Table 6 and the ESI). Interestingly, moving from neat water to urea concentration of 9 M the static permittivity of aqueous urea solutions increases from 78.4 to nearly 102 at 25 °C, i.e., a static permittivity nearly as high as that of formamide [52]. This marked dielectric properties alteration is presumably associated with the formation of H-bonded clusters of urea and unbound water molecules resulting in highly dipolar species contributing to a high static permittivity. Different researchers have concluded via theoretical and experimental observations that urea hardly breaks the structure of water and this holds true both in low concentration urea solutions as well as high.

In terms of the polarity characteristics of aqueous urea solutions, apart from the aforementioned drastic increase in permittivity observed when increasing urea concentration, a milder yet sizable increase in the refractive index is also observed (see Table 6). However, using polarity parameter *f*(*x*) (see Section 4 for details) the increase in dipolarity of aqueous urea as urea’s concentration increases (expressed through function *f*(ϵ)) is more subtle compared to the observed increase in polarizability expressed through *f*(*n*^2^); see the ESI for details). In turn, Dimroth–Reichardt’s polarity scale decreases upon the increase in urea’s concentration from 63.1 kcal/mol for neat water to 61.2 kcal/mol at a urea concentration of 500 mg/g (approx 9.5 M; see Table 6 for more data and the ESI for corresponding plots). The fact that *E_T_*(30) is a measure of Lewis acidity and dipolarity/polarizability of the medium the above-described observations on *f*(ϵ), *f*(*n*^2^), and *E_T_*(30) indicate a significant decrease in Lewis acidity with an increase in urea concentration. Additionally, another solvatochromic compound namely dicyanobis(1,10-phenanthroline)iron(II) (**7**) has been shown to be responsive to urea in a concentration-dependent fashion [29] but of low sensitivity (*vide infra*). The observed response of **7** was recorded as a hypsochromic effect in its visible spectra (specifically the MLCT band) upon increasing urea concentration [29].

**Figure 3:**
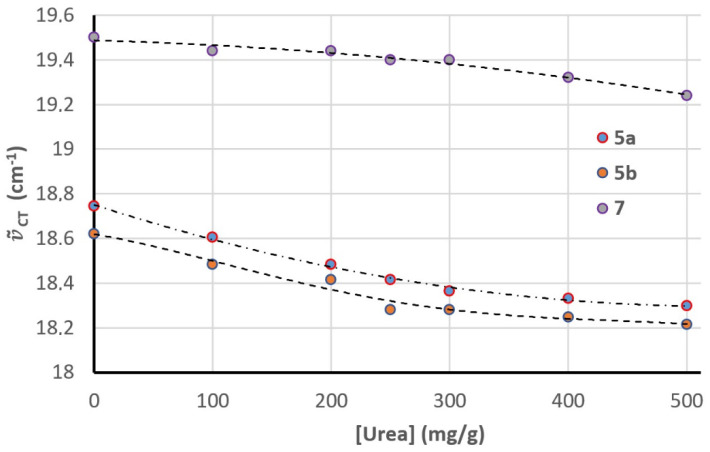
Plots of measured ${\tilde{v}}_{CT}$ of solvatochromic compounds **5a**, **5b**, and **7** against urea concentration. Lines in all cases are polynomial fits added for visualization purposes.

**Figure 4:**
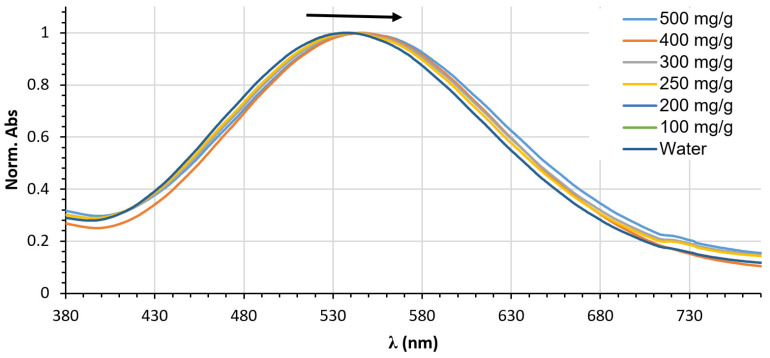
Partial normalized UV-Vis spectra of **5a** in water at varying urea concentrations (0 to 9.48 M). The absorbance has been normalized to a value of 1 (Arrow represents bathochromic shift of the MLCT-band upon increase of urea concentration).

Taking into account these findings on aqueous urea, the presented study focuses both on dilute solutions lower than 2 M as well as solutions with urea concentration up to approx. 9.5 M. Notably, both compounds exhibited a mild response to polarity changes induced when increasing urea’s concentration (average 47.5 cm^−1^/M for **5a** and 35.9 cm^−1^/M for **5b**; see Table 7 and Figure 3 and Figure 4). Notably, the average sensitivity of complex **7** is 24.4 cm^−1^/M i.e., nearly half of the recorded one for **5a** and siginficantly lower than that of **5b**. The latter finding signifies the superiority of the studied complexes towards sensing polarity changes in aqueous urea. Taking into account the positive solvatochromic response of **5a** and **5b** to the increase in f(*ϵ*) or *f*(*n*^2^) i.e., bathochromism upon the increase in dipolarity/polarizability the question that arises is “*Is there an inversion of solvatochromism when moving from molecular solvents to aqueous urea?*”.

### 2.7. An Inverted Solvatochromic Effect or a Pronounced Specific Effect?

**Figure 5:**
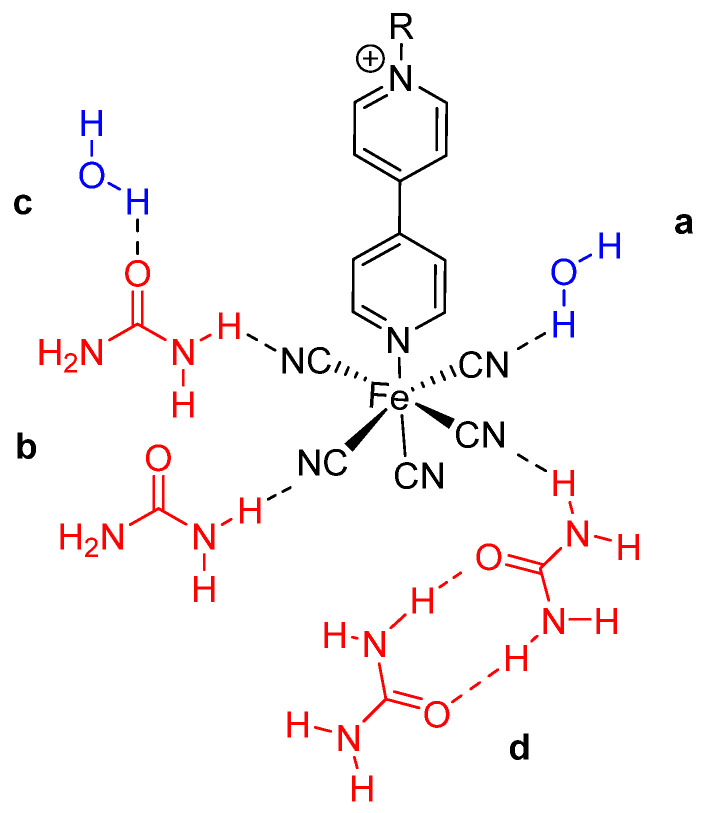
Plausible H-bonding motifs encountered in aqueous urea solutions of to **5a** and **5b** with low to intermediate urea concentration (<5 M) (a, water molecule (blue); b, urea molecule (red); c, water–urea dimer; d, urea dimer). R, substituent representing the remaining, non-displayed part of **5a**/**5b**.

Answering this question is not trivial since it is not clear which type of interaction dominates at different urea concentrations. An FTIR study was conducted to complement this analysis. This study shows small shifts and slight broadening of the C≡N stretching bands of **5ab** when varying urea concentration from 0 to 9.48 M (see Figure S6, Supporting Information File). The shift in reference to water solution was as anticipated more obvious yet a clear shift was observed while increasing the concentration of urea in the aqueous phase (see Figure S6, Supporting Information File). The results for both **5a** and **5b** were very similar. Small but noticeable shifts in the C≡N stretching band of both compounds were observed while increasing the concentration of urea solutions. This effect is associated with the development of hydrogen bond interactions between an HBD molecule and the CN groups of a cyanoferrate. The aforementioned H-bonding interaction can cause a shift in the vibrational frequency of the CN stretching mode. This shift can be observed as a blue shift in the infrared or Raman spectra and as anticipated, the extent of this shift depends on the strength of the hydrogen bond and the nature of the hydrogen bond donor and acceptor [57]. In the case of **5ab**, it was observed that, regardless of the concentration increase, no further shift in the C≡N stretching bands was detected when there was an excess of urea. Once completed, hydrogen bonding between the CN-groups of **5ab** and urea is achieved, any further changes in the dielectric properties of the solution have no significant effect on the C≡N stretching bands of the pentacyanoferrates. It is worth noting that while the effects observed through FTIR are valuable, they were quite subtle and not significantly above the instrument’s sensitivity threshold. Understanding the hydrogen bonding interactions among urea, water, and their complexes with pentacyanoferrates remains challenging and falls outside the scope of this study. However, based on the background analysis (*vide supra*), the author proposes potential hydrogen bonding motifs, as illustrated in Figure 5.

### 2.8. Setting a Suitable Equation Describing Aqueous Urea Polarity Sensing

A good way to represent the observed aqueous urea polarity sensing mathematically is through an equation involving parameters expressing key medium polarity properties. Unfortunately, there are no theoretical properties describing the acidity of such solutions, in contrast to dipolarity and polarizability [58]. In light of this, a hybrid equation involving the empirical *E_T_*(30) parameter by Reichardt and the functions *f*(ϵ) and *f*(*n*^2^) is employed (see Equation (10)). Equation (10) attempts to represent the MLCT maxima wavenumbers ${\tilde{v}}_{CT}$ in a very specific manner. It does so by subtracting dipolarity and polarizability from the empirical parameter *E_T_*(30) through the functions *f*(ϵ) and *f*(*n*^2^), thus removing their proportional contribution for the given medium (aqueous urea). The sensitivities *m*_2_ and *m*_3_ and the intercept *m*_1_ are determined through regression using the experimental ${\tilde{v}}_{CT}\;$data (Table 7) and the corresponding values of *E_T_*(30), *f*(ϵ), and *f*(*n*^2^), at different urea concentrations (see Table 6; also for more details on the methodology see Section 4).

Practically, Equation (10) provides ${\tilde{v}}_{CT}$ as a function of Lewis acidity since the proportion of dipolarity that *E_T_*(30) imparts is removed by subtracting the function *f*(ϵ) − *f*(*n*^2^) expressing dipolarity of the medium. The results of Equation (10) for 5a and 5b are included in Table 8.

**Figure 6:**
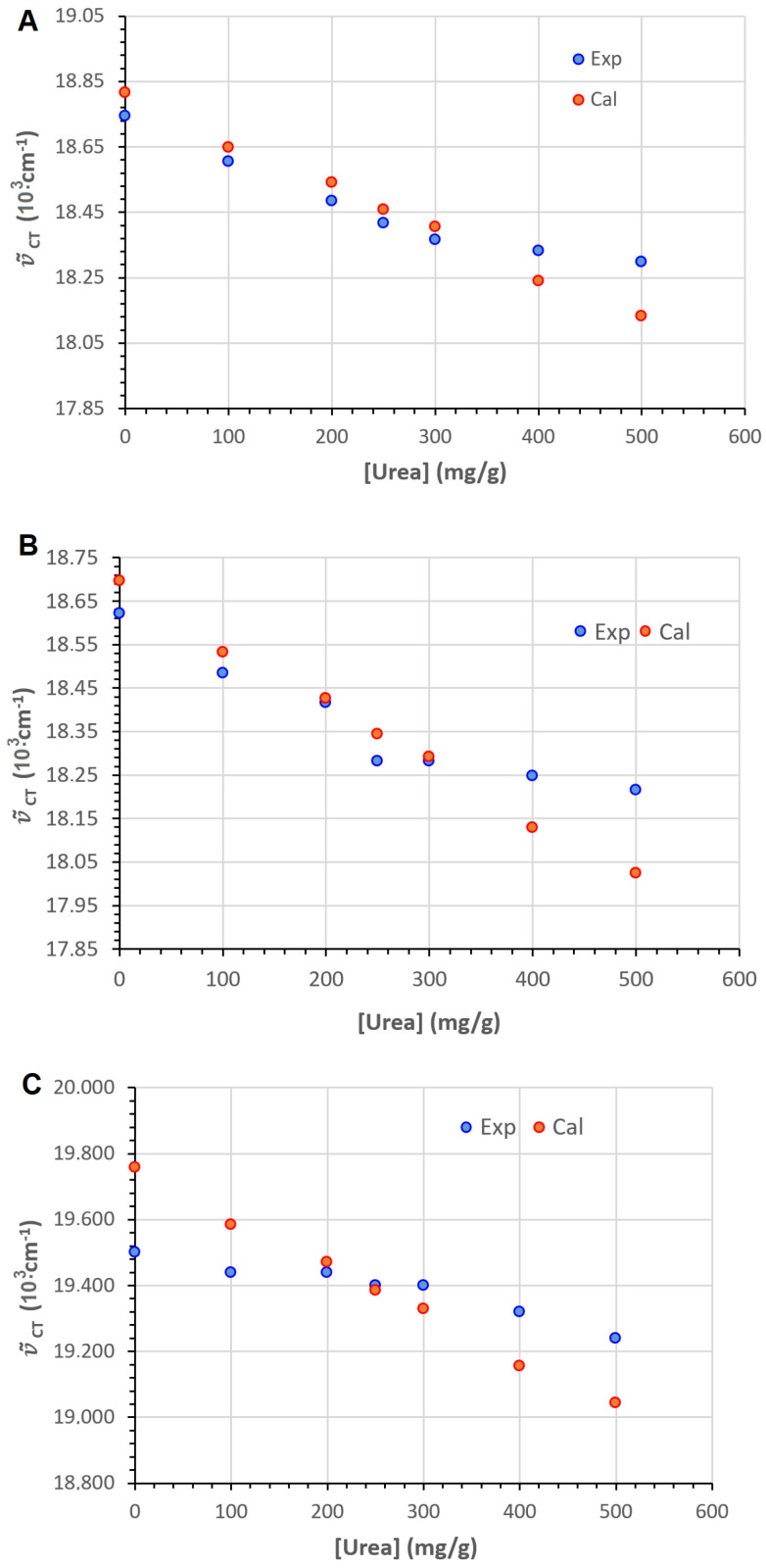
Experimental and calculated through Equation (10) MLCT maxima wavenumbers (${\tilde{v}}_{CT})$ values for solvatochromic compounds (**A**) **5a**, (**B**) **5b** and (**C**) **7**.

The obtained *m*_1_, *m*_2_ and *m*_3_ values provide accurate predictions of the ${\tilde{v}}_{CT}$ for both compounds as implied by the high regression coefficients of the linear fitting of experimental and calculated ${\tilde{v}}_{CT}$ values (see Figure 6). The model’s prediction of a positive dependency of the ${\tilde{v}}_{CT}$ on *E_T_*(30) aligns with the assumption of a more stabilizing environment for the MLCT excited state of the compounds in aqueous urea when increasing urea concentration. This increase is associated with a decrease in Lewis acidity, as expressed by the *E_T_*(30) parameter. Additionally, the relative importance of *E_T_*(30) in the observed solvatochromism of both compounds was found to be the highest, around 60% in both cases (see Table 8) being slightly lower in the case of compound 5b. The negative sensitivity of ${\tilde{v}}_{CT}$ to *f*(ϵ) − *f*(*n*^2^) observed in both cases (since *m_3_* was positive in both cases; Table 8 and Equation (10)), along with the very low contribution of dipolarity (expressed via *f*(ϵ) − *f*(*n*^2^)), suggests that the phenomenon should not be attributed to an inverted solvatochromism. Instead, it potentially indicates the dominance of specific interactions, particularly hydrogen bonding, in the cybotactic region involving compounds **5a** and **5b** as solutes.

On the other hand, Compound **7** (a dicyanoferrate(II) solvatochromic complex) exhibited qualitatively the same behavior as probes **5a** and **5b**, i.e., **a** decrease of ${\tilde{v}}_{CT}$ (hypsochomism) upon increasing urea concentration. Nonetheless, the sensitivity of **7** to the solvent polarity changes due to the influence of the increase in urea concentration on the medium, was lower than any of the compounds **5a** or **5b** (6 nm for **7**, 13 nm, and 12 nm, respectively, for **5a** and **5b** within the range of urea concentration 0 and 9.48 M, respectively). This finding underlies their superiority over this renowned solvatochromic compound. Model Equation (10) was not as successful in describing the solvatochromic effect for **7** as compared to **5a** and **5b** (see Table 8 and Figure 6 for details).

## 3. Conclusions

Two intensely solvatochromic pentacyanoferrate(II) complexes were synthesized and characterized, representing a significant advancement over existing cyanoferrate(II) compounds. Indeed, high solvatochromic shifts were consistently observed for both compounds, surpassing in some cases (e.g., water–NMF difference) even those of the reference betaine dye, compound **6**. Their solvatochromism was examined in various solvents, including protic and non-protic solvents, as well as binary mixtures of molecular solvents. A strong negative solvatochromic effect was observed, with slight differences between the two compounds attributed to the structural diversity of the ligands. The rationalization of solvatochromic shifts in terms of solute–solvent interactions revealed a strong dependency on Lewis acidity and the dipolarity/polarizability of the medium. This analysis, based on a well-defined set of LSERs, provided complementary insights into specific and non-specific solute–solvent effects. Moreover, dipole moments in the excited and ground states were determined using the Suppan–Tsiamis method based on spectral data. It was found that compound **5b** exhibits a more polar excited state and a larger dipole moment difference between MLCT excited and ground state compared to compound **5a**. This finding was attributed to the differing side-heterocycles between the two compounds. The examination of the solvatochromism of **5a** and **5b** and their sensitivity to urea concentration in aqueous urea revealed that both complexes exhibit higher sensitivity compared to the model complex **7**, a model cyanoferrate(II) solvatochromic model. Compounds **5a** and **5b** demonstrated the capacity to probe significant changes in Lewis acidity upon increasing urea concentration, though with a relatively lesser impact of dipolarity on their solvatochromic sensitivity. A newly developed LSER model was used to evaluate whether inverted solvatochromic effects or specific solute–solvent interactions govern the observed behavior in urea-containing solutions. The results indicated that in both cases, the observed solvatochromic phenomena are largely governed by specific solute–solvent effects, and the inverted solvatochromism hypothesis can be ruled out. The new LSER model was further validated by predicting the maxima MLCT wavenumbers for both compounds, as well as for the reference model compound **7**, yielding satisfactory results.

In summary, this work highlights the unique solvatochromic aptitude and urea sensing capacity of two relative pentacyanoferrate(II) complexes, showcasing their ability to engage in diverse solvent–solute interactions and deliver valuable insights into medium polarity. These findings can contribute to a deeper understanding of solute–solvent dynamics and enhance methodologies for polarity assessment in both organic solvents and complex aqueous mixtures.

## 4. Materials and Methods

### 4.1. Materials

All compounds/reagents were purchased from Sigma Aldrich, Stockholm, Sweden. All solvents were HPLC-grade and they were purified prior to use, according to literature [59]. Water was purified with a Barnstead EASYpure RF compact ultrapure water system and then distilled twice.

### 4.2. Model Compounds

**Figure 7:**
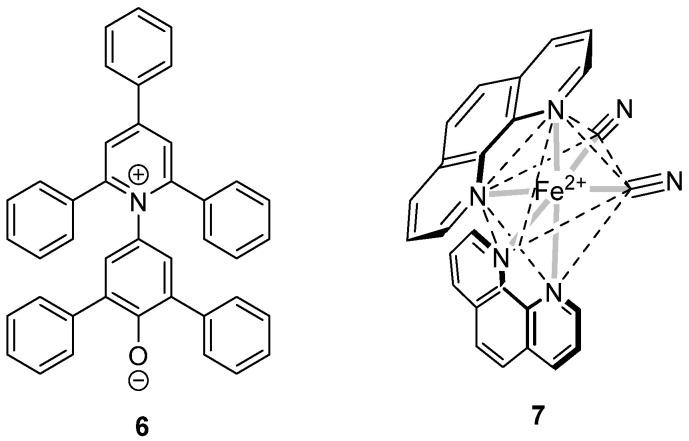
Reference solvatochromic compounds.

Two model solvatochromic compounds were used in this study for the rationalization of the observed solvatochromic shifts of **5a** and **5b**. Compound **6**, the renowned Reicharst’s solvatochromic betaine the solvatochromic shifts which are the basis of the polarty scale *E_T_*(30) [39] and compound **7** (see Figure 7), a neutral phenanthroline ligand bearing dicyanoferrate(II) complex with intense solvatochromic responsiveness [39]. The structure of the latter resembles a lot to that of compounds **5a** and **5b** and serves as a good model for rationalization of the cynoferrates(II)-solvent interactions.

### 4.3. Computations

#### 4.3.1. Linear Solvation Energy Relationships (LSERs)

In the context of the study of correlations of ${\tilde{v}}_{CT}$ with different solvent polarity parameters the following mono- and multi-parametric LSERs were employed as follows:

(1) $${{\tilde{v}}_{CT}=\tilde{v}}_{o}+e\cdot E_{T}^{N}$$

(2) $${{\tilde{v}}_{CT}=\tilde{v}}_{o}+s\cdot \pi^{*}+a\cdot \alpha +b\cdot \beta$$

(3) $${{\tilde{v}}_{CT}=\tilde{v}}_{o}+s\cdot \pi^{*}+a\cdot \alpha$$

(4) $${{\tilde{v}}_{CT}=\tilde{v}}_{o}+e\cdot E_{T}^{N}+b\cdot \beta$$

Equation (1) correlates ${\tilde{v}}_{CT}$ with the normalized polarity scale by Reichardt [39], Equation (2) is the so-called Kamlet–Taft–Abboud (KAT) equation [44] and Equation (3) its reduced form excluding parameter β [19].

Equation (4) is analogous to Krygowski–Fawcett equation which is often used to quantify and rationalize the effects of solvent polarity on a variety of physicochemical properties and spectroscopic data [46]. In its original form the model employs Dimroth–Reichardt *E_T_*(30) parameter and the Gutmann’s donor numbers DN. In fact, we previously used this equation encompassing normalized parameters (i.e., normalized Reichardt solvent polarity scale and *DN^N^* (normalized donor number) yet, here it is employed with parameter *β* instead of *DN^N^* [60]. One of the benefits of using the normalized versions is that both parameters involved are dimensionless and very within the same range, i.e., 0 to 1. More details can be found in a recent publication where Equation (4) was utilized to rationalize solvatochromic data in binary solvent mixtures [14].

All linear correlations (both mono- and multi-parametric) were performed using *RStudio* (ver: 2024.09.1+394). The calculations included the determination of standard errors for the parameters, as well as the calculation of the residual sum of squares (*rss*) and the coefficient of determination (*r*^2^). The RStudio environment provided the necessary tools and packages to accurately perform these statistical analyses.

#### 4.3.2. Dipole Moment Calculations

For the determination of dipole moments in ground and MLCT excited states of compounds **5a** and **5b** the model by Suppan and Tsiamis [50] (Equation (5)) was used. The model is ideal for dyes that are not emissive.

(5) $$E_{CT}=\frac{1}{4\pi \varepsilon_{o}}\left[\frac{\vec{\mu_{g}}\cdot \left(\vec{\mu_{g}}-\vec{\mu_{e}}\right)}{{a_{r}}^{3}}\left(\varphi \left(\epsilon \right)-\varphi \left(n^{2}\right)\right)+\frac{\left(\mu_{e}^{2}-\mu_{g}^{2}\right)}{{a_{r}}^{3}}\varphi \left(n^{2}\right)\right]$$

(6) $$\varphi \left(x\right)=\frac{2(x-1)}{2x+1}$$

The cavity terms (Van der Waals radii) of both compounds $(a_{r}$(Å)) were determined using the following procedure: First, the structures were drawn in *Chemtool* 1.6.15 and then optimized using Molecular Mechanics on *Avogadro* 1.2.0. The UFF force field was used in both cases. The resulting configurations were further used as input for the determination of the Van der Waals radii using 3V:VossVolume Voxalator, available online. The latter was directly used as the cavity term.

#### 4.3.3. Determination of Relative Importance (Contribution) of Each of the Parameters Involved in the LSERs

For calculating relative importance (contribution) of each of the parameters involved in the LSERs and other equations (Equations (1)–(4) and (10)), the following methodology was employed. A general multiparametric LSER model can be assigned as in the general Equation the quantity Q with the parameters X_1_, …, X_i_, …, X_n_, according to Equation (3) the following is assumed [60]:

(7) $$Q=Q_{o}+\sum_{i=1}^{n}\left(x_{i}X_{i}\right)$$

(8) $$P\left(X_{i}\right)=\frac{100x_{i}^{\prime }}{\sum_{i=1}^{n}x_{i}^{\prime }}$$

where

(9) $$x_{i}^{\prime }=\left|x_{i}\right|\sqrt{\frac{\sum_{j=1}^{m}(X_{ij}-\bar{X_{i}})}{\sum_{j=1}^{m}(Q_{j}-\bar{Q})}}$$

In this equation, Q_o_ is the regression value of the intercept of this linear model, and x_1_, …, x_i_, …, x_n_ are the coefficients of the parameters X_1_, …, X_i_, …, X_n_, respectively. These coefficients reflect the sensitivity of the quantity Q to the parameters X_1_, …, X_i_, …, X_n_. The coefficients x_1_, …, x_i_, …, x_n_ are obtained through linear multiparametric regression analysis. In this work, n = 3 for Equation (2); n = 2 for Equations (3) and (4). Furthermore, assuming that we have *m* series of data, e.g., *m* different cases of solvents (in the case of this work *m* = 9 solvents), the relative contribution of each one of the parameters X_i_ [symbolized as P(X_i_)] to the quantity Q, can be calculated through Equation (8). In Equation (8), (x_i_)′ is calculated through Equation (9), where $\bar{X_{i}}$ and $\bar{Q}$ are the mean values of the parameter X_i_ and of the quantity Q, respectively, and finally |x_i_| is the absolute regression value of x_i_ obtained from the regression analysis.

#### 4.3.4. Model Describing Aqueous Urea Solvatochromic Sensing

(10) $${\tilde{v}}_{CT}=m_{1}+m_{2}E_{T}\left(30\right)-m_{3}\left(f\left(\epsilon \right)-f\left(n^{2}\right)\right)$$

$with\;{{m_{1},\;m}_{2},\;m}_{3}>0$

and

(11) $$f\left(x\right)=\frac{x-1}{2x+1}$$

from Kirkwood–Bauer–Mataga (KBM) equation [39].

Description of the method: The R package *nloptr* (Version 2.1.1) was used to perform the optimization (see Equation (10)). An objective function was constructed to minimize the sum of squared residuals between the observed and predicted values. The function was defined to include the parameters of interest and initial values for these parameters were set to c(1,1,1). The lower and upper bounds for the parameters were defined as c(0,0,0) and c(∞,∞,∞), respectively, in order to ensure that ${{m_{1},\;m}_{2},\;m}_{3}>0$. The optimization was performed using the *NLOPT_LN_COBYLA* algorithm with a relative tolerance of 1 × 10^−6^. The optimized parameters ${{m_{1},\;m}_{2},\;m}_{3}$ were extracted from the result object obtained after the optimization process (are listed in Table 8).

#### 4.3.5. Spectroscopic and Analytical Methods

NMR spectra were obtained using a Varian Gemini 300 spectrometer (300 MHz ^1^H, 75 MHz ^13^C). Both ^1^H and ^13^C NMR spectra were recorded in DMSO-d6 at 25  ±  1 °C. The 1H NMR spectra were calibrated by using residual undeuterated solvent as an internal reference (4.79 ppm) and ^13^C NMR spectra were calibrated using the DMSO-d6 signal at 39.52 ppm [61]. Abbreviations are used for multiplicity in the text: s = single; d = doublet; m = multiplet; arom = aromatic; Ph = phenyl.

UV-Vis spectra were recorded using a Varian CARY 1E UV–Vis spectrophotometer, Palo Alto, CA, USA. Regarding the solvatochromism of compound 4, typical solutions with a concentration of 750 ppm (approx. 1 mM) were prepared right before any measurement, and measured at 25 ± 1 °C. Each measurement was repeated three times; therefore, each of the values of CT energies listed in Table 2 and Table 7 correspond to the average of three measurements (standard deviation 0.5 nm).

Fourier-transform infrared spectroscopy: Infrared spectrum were recorded using a Fourier-transform infrared spectrophotometer (Spectrum Two, Perkin-Elmer, Llantrisant, UK) equipped with a Universal Attenuated Total Reflectance diamond. All FTIR spectra were collected at a spectrum resolution of 4 cm^−1^, with 32 scans from 4000 to 500 cm^−1^.

Thermogravimetric analysis: Thermograms were made with a Mettler-Toledo TGA2 (Mettler Toledo, Greifensee, Switzerland), under nitrogen with a flow rate of 40 mL min^−1^, using alumina pans. 5 to 10 mg of each sample were put in a standard TGA alumina crucible pan at a heating rate of 10 °C min^−1^.

Elemental Analyses were performed on a Perkin-Elmer Elemental Analyzer 2400 CHN, MA, USA.

### 4.4. Syntheses

#### 4.4.1. General Method for the Synthesis of Products (**3a-b**)

In a solution of α,α′-dichloro-*p*-xylene (**1**) (171 mg, 1 mmol in 5 mL CHCl_3_), half the stoichiometric amount (0.5 mmol) of the heterocyclic compound (**2a-b**) was added. The solution was then left to boil under stirring for 10 h, during which a white precipitate formed. This precipitate was filtered under vacuum and washed with CHCl_3_ and then with diethyl ether. The solids were dried under vacuum for several hours and stored in a vacuum desiccator in the presence of P_2_O.

#### 4.4.2. 2-(4-(Chloromethyl)benzyl)isoquinolin-2-ium (**3b**)

White hygroscopic solid, 118 mg, 78%, mp > 250 °C. ^1^H NMR (300 MHz, D_2_O): δ = 9.81 (s, 2H, isoq.), 8.56 (d, *J* = 6.3 Hz, 2H, isoq.), 8.39 (m, 3H, arom.), 8.17 (m, 3H, arom.), 7.98 (t, *J* = 5.7 Hz, 1H, isoq.), 7.65 (s, 2H, (-CH_2_)-isoq.), 6.00 (s, 2H, (-CH_2_)-Cl). ^13^C NMR (75 MHz, D_2_O/[D6]DMSO): 150.95, 139.25, 139.02, 136.38, 135.56, 133.13, 131.81, 131.56, 129.31, 128.82, 128.35, 65.35 (-(CH_2_)-py), 40.09 (-(CH_2_)-Cl).

#### 4.4.3. Preparation of Products (**4a-b**)

Product **3a-b** (0.427 mmol) was dissolved in 5 mL of DMF. Addition of 4,4′-Dipyridine: An excess amount of 4,4′-dipyridine (135 mg, approximately 0.855 mmol, which is 2 times the amount of **3a-b**) was added to the solution. The solution was placed in an oil bath at a temperature of 100–110 °C for 10 h. A total of 20 mL of anhydrous acetone was added to the solution, and immediately the product (**4a-b**) was formed as a white precipitate. The solid was filtered with suction and washed several times with ethanol (to remove DMF) and diethyl ether. Finally, the product was dried under vacuum for several hours. Both products (**4a-b**) are highly hygroscopic and should be stored in a vacuum desiccator with P_2_O_5_.

#### 4.4.4. 2-(4-([4,4′-Bipyridin]-1-ium-1-ylmethyl)benzyl)isoquinolin-2-ium: Ligand: Ligand **4b**

Off-white hygroscopic powder, 140 mg, 72%, mp > 250 °C. ^1^H NMR (300 MHz, D_2_O): δ = 9.44 (d, J = 6.9 Hz, 2H, C_5_H_4_N), 9.36 (d, J = 6.9 Hz, 2H, C_5_H_4_N), 8.84 (d, J = 6.6 Hz, 2H, C_5_H_4_N), 8.75 (d, J = 7.2 Hz, 2H, C_5_H_4_N), 8.63 (d, J = 6.9 Hz, 1H, C_5_H_4_N), 8.55 (d, J = 6.9 Hz, 1H, isoq.), 8.42 (d, J = 6.3 Hz, 1H, isoq.), 8.10 (d, J = 6.3 Hz, 2H, isoq.), 7.99 (d, J = 6.9 Hz, 2H, C_5_H_4_N), 7.11 (d, J = 7.2 Hz, 4H, Ph), 5.93 (s, 4H, CH_2_).13C NMR (75 MHz, D_2_O/[D6]DMSO): 152.32, 149.85, 146.18, 144.89, 144.32, 142.77, 140.17, 131.42, 130.56, 128.92, 128.00, 127.36, 127.21, 126.23, 124.67, 121.39, 62.4 (CH_2_), 61.9 (CH_2_).

#### 4.4.5. General Method for the Synthesis of Solvatochromic Products (**5a-b**)

In an aqueous solution of the heterocyclic ligand (**5a-b**) (0.450 mmol in 5 mL H_2_O), 0.500 mmol (163 mg) of freshly prepared Na_3_[Fe^II^(CN)_5_NH_3_]·3H_2_O salt was added. The solution immediately took on a characteristic violet color (due to complex formation). The solution was stirred in the dark under an inert atmosphere (Ar) at room temperature for 6 h. Then, ethanol (EtOH) was added in a volume six times that of the solution (30 mL) and the mixture was left in the dark at 4 °C for 12 h, during which a colored precipitate formed in all cases. The precipitate was collected by vacuum filtration, and washed sequentially with cold EtOH and diethyl ether (Et_2_O) several times. Finally, the product was dried under vacuum at a temperature above 40 °C.

#### 4.4.6. Solvatochromic Complex **5b**

Deep blue solid, 237 mg (0.305 mmol) 68% mp> 300 °C (dec.). ^1^H NMR (300 MHz, D_2_O): 9.52 (d, J = 6.9 Hz, 2H, C_5_H_4_N), 9.42 (d, J = 6.9 Hz, 2H, C_5_H_4_N), 8.90 (d, J = 6.9 Hz, 2H, C_5_H_4_N), 8.83 (d, J = 6.9 Hz, 2H, C_5_H_4_N), 8.69 (d, J = 7.2 Hz, 1H, C_5_H_4_N), 8.59 (m, 2H, isoq.), 8.16 (d, J = 6.9 Hz, 2H, isoq.), 8.07 (d, J = 7.2 Hz, 2H, C_5_H_4_N), 7.21 (d, J = 9.2 Hz, 4H, Ph), 5.99 (s, 4H, CH_2_). ^13^C NMR (75 MHz, D_2_O/[D_6_]DMSO): 172.34 (CΝ), 152.96, 150.15, 146.83, 144.99, 144.82, 143.17, 140.53, 132.02, 130.86, 129.12, 128.66, 127.82, 127.91, 126.79, 124.78, 121.94, 62.69 (CH_2_), 62.81 (CH_2_). Elemental analysis calcd (%) for: C_32_H_35_FeO_6_NaN_8_ (C_32_H_23_FeNaN_8_·6H_2_O) C: 54.40%, H: 4.99%, N: 15.86%; found: C: 54.73%, H: 5.26%, N: 15.58%; ΤGA: loss of approx. 6 molecules of H_2_O.

The synthesos of compounds **3-5a** have been reported earlier by the author [18].

## Figures and Tables

**Table 1:** Solvents and solvent mixtures used for this study of solvatochromism of **5a** and **5b** and the corresponding solvent polarity parameters and functions ^♠^.

Solvent	*α* [39]	*β* [39]	π* [39]	*E_T_*(30) [39] (kcal/mol)	$E_{T}^{N}$ ^■^	*ϵ* [40]	*n* [41]	*φ*(*ϵ*) ^†^	*φ*(*n*^2^) ^†^
HOH	1.17	0.47	1.09	63.1	1.000	78.5	1.3325	0.9810	0.3408
TFE	1.51	0.00	0.73	59.8	0.898	26.7	1.2907	0.9449	0.3074
Glycerol	1.14	0.87	0.92	57.0	0.812	42.5	1.4746	0.9651	0.4391
^‡^ EtOH50%	1.10	0.54	0.96	56.5	0.796	38.1	1.3617	0.9611	0.3628
EG	0.90	0.52	0.92	56.3	0.790	41.4	1.4318	0.9642	0.4118
FA	0.71	0.48	0.97	55.8	0.775	109.5	1.4475	0.9864	0.4220
MeOH	0.98	0.66	0.60	55.4	0.762	33.8	1.3264	0.9563	0.3361
^‡^ AcMe50%	0.96	0.47	1.00	54.9	0.747	30.0	1.3583	0.9508	0.3603
NMF	0.62	0.80	0.90	54.1	0.722	182.4	1.4319	0.9918	0.4118

^♠^ Solvents are displayed in decreasing *E_T_*(30) mode (top to bottom). † Calculated using Equation (6) with *x* = ϵ  for φ(ϵ) and *x* = $n^{2}$ for $\varphi (n^{2})$. ‡ Determined through the interpolation method described in ref. [14]. **^■^** Normalized Reichardt’s solvent polarity scale [39].

**Table 2:** Measured MLCT wavelength maxima of **5a** and **5b**, $\lambda_{CT}^{5a}$ and $\lambda_{CT}^{5b}$ respectively, in different solvents and the corresponding MLCT wavenumbers, ${\tilde{v}}_{CT}^{5a}$ and ${\tilde{v}}_{CT}^{5b}$.

Solvent	$\lambda_{CT}^{5a}$ (nm) *	$\lambda_{CT}^{5b}$ (nm) ^‡^	${\tilde{v}}_{CT}^{5a}$$\;(10^{3}\;{\;cm}^{-1}$)	${\tilde{v}}_{CT}^{5b}$$(10^{3}\;{\;cm}^{-1}$)
HOH	534	537	18.744	18.622
TFE	572 ^†^	577	17.470	17.341
Glycerol	605	610	16.529	16.389
EtOH50%	585	585	17.094	17.094
EG	600	599	16.667	16.705
FA	628	638	15.923	15.664
MeOH	671	690	14.903	14.497
AcMe50%	599	604	16.694	16.564
NMF	626	625	15.978	16.009

* Data reported previously by [18]. ^†^ Measurements in TFE have not been previously reported for **5a**. ^‡^ Results reported in this study.

**Table 3:** Intercepts ${\tilde{v}}_{o}$ and coefficients of parameters $E_{T}^{N}$, π*, a, and *β* (*e, s, a* and *b, respectively*) determined through regression analysis using LSERs 1–4 for **5a** and **5b**. # corresponds to number.

Solvent Parameter/Correlation Coefficient							
Compound/Equation #		$E_{T}^{N}$	π*	*a*	*β*		
	${\tilde{v}}_{o}$(10^3^·cm^−1^)	*e*(10^3^·cm^−1^)	*s*(10^3^·cm^−1^)	*a*(10^3^·cm^−1^)	*b*(10^3^·cm^−1^)	*r* ^2^	*rse*
**5a**/1	8.119 ± 2.064	10.54 ± 2.531	-	-	-	0.712	0.618
**5a**/2	9.547 ± 1.636	-	5.486 ± 1.151	9.547 ± 1.636	−0.730 ± 0.839	0.881	0.470
**5a**/3	8.733 ± 1.314	-	5.487 ± 1.127	2.972 ± 0.630	-	0.864	0.460
**5a**/4	8.594 ± 2.833	10.14 ± 3.090	-	-	−0.288 ± 1.072	0.716	0.664
**5b**/1	8.121 ± 2.525	10.39 ± 3.096	-	-	-	0.617	0.756
**5b**/2	9.052 ± 1.971	-	5.958 ± 1.387	2.509 ± 0.968	−0.731 ± 1.012	0.847	0.566
**5b**/3	8.238 ± 1.551	-	5.959 ± 1.330	2.928 ± 0.743	-	0.831	0.543
**5b**/4	8.513 ± 3.477	10.06 ± 3.792	-	-	−0.238 ± 1.315	0.619	0.815

**Table 4:** Relative importance of parameters $E_{T}^{N},\;\pi$*, α,   and β ($R_{E_{T}^{N}},\;R_{\pi *},\;R_{\alpha },\;R_{\beta }\;$, respectively) in LSERs 2, 3, and 4 for compounds **5a** and **5b**. # corresponds to Equation number.

Compound/Equation #	$R_{E_{T}^{N}}$	$R_{\pi *}$	$R_{\alpha }$	$R_{\beta }$
**5a**/2	-	0.52	0.34	0.14
**5a**/3	-	0.52	0.48	-
**5a**/4	0.85	-	-	0.15
**5b**/2	-	0.58	0.29	0.13
**5b**/3	-	0.58	0.42	-
**5b**/4	0.86	-	-	0.14

**Table 5:** Results of application of Suppan-Tsiamis model in **5a** and **5b**.

	$a_{r}$(Å)	$k_{c}^{*}\left[\frac{\vec{\mu_{g}}\cdot \left(\vec{\mu_{g}}-\vec{\mu_{e}}\right)}{{a_{r}}^{3}}\right]\;(J)$	$k_{C}\left[\frac{\left(\mu_{e}^{2}-\mu_{g}^{2}\right)}{{a_{r}}^{3}}\right](J)$	$\left\|\vec{\mu_{g}}\right\|$ (D)	$\left\|\vec{\mu_{e}}\right\|\;(D)$	*r* ^2^	${rse\;}^{■}$
**5a**	5.01	$4.408\cdot 10^{-19}\pm \;7.106\cdot 10^{-20}$	$\;\;\;\;\;1.869\cdot 10^{-19}\pm \;1.107\cdot 10^{-19}$	15.1	21.5	0.997	$2.168\cdot 10^{-20}$
**5b**	5.03	$\;\;\;\;\;4.263\cdot 10^{-19}\pm \;7.538\cdot 10^{-20}$	$\;\;\;\;\;2.044\cdot 10^{-19}\pm \;1.174\cdot 10^{-19}$	14.8	21.9	0.996	$2.299\cdot 10^{-20}$

$k_{c}^{*}:\;Coulomb’s\;constant;\;k_{C}=\frac{1}{4\pi \varepsilon_{o}}≅8.988\cdot 10^{9}\;Nm^{2}C^{-2}$. ^■^ *rse*: residual standard error (see details in Section 4).

**Table 6:** Physicochemical and polarity properties of the studied aqueous urea solutions.

Urea Conc. (mg/g)	ρ (g/L) [52]	[Urea] (mol/L)	*E_T_*(30) [29] (kcal/mol)	${\tilde{v}}_{CT}^{7}$$\;[29]\;(10^{3}{\;cm}^{-1}$)	ϵ [52]	n [56]	f(ϵ) ^♠^	$f(n^{2})$ ^♠^
0	998.68	0.00	63.1	19.50	78.48	1.3310	0.4905	0.1706
100	1023.4	1.70	62.5	19.44	83.74	1.3472	0.4911	0.1760
200	1049.7	3.50	62.1	19.44	87.76	1.3621	0.4915	0.1816
250	1063.5	4.43	61.8	19.40	90.10	1.3698	0.4917	0.1844
300	1077.7	5.38	61.6	19.40	92.48	1.3778	0.4919	0.1873
400	1107.2	7.37	61.4	19.32	96.45	1.3943	0.4923	0.1931
500	1138.3	9.48	61.2	19.24	102.1	1.4118	0.4927	0.1992

**^♠^** General Polarity function: $f\left(x\right)=\frac{x-1}{2x+1}.$

**Table 7:** MLCT wavelength maxima: $\lambda_{CT}^{5a}$ and $\lambda_{CT}^{5b}$ and corresponding wavenumbers ${\tilde{v}}_{CT}^{5a}$ and ${\tilde{v}}_{CT}^{5b}$ determined in aqueous urea solutions of compounds **5a** and **5b**, respectively.

Urea Concentration (mg/g)	$\lambda_{CT}^{5a}$ (nm)	${\tilde{v}}_{CT}^{5a}\;(10^{3}{cm}^{-1})$	$\lambda_{CT}^{5b}$ (nm)	${\tilde{v}}_{CT}^{5a}\;(10^{3}{cm}^{-1})$
0	533.5	18.744	537.0	18.622
100	537.5	18.605	541.0	18.484
200	541.0	18.484	543.0	18.416
250	543.0	18.416	547.0	18.282
300	544.5	18.365	547.0	18.282
400	545.5	18.332	548.0	18.248
500	546.5	18.298	549.0	18.215

**Table 8:** Results of application of the model Equation (10) in **5a** and **5b**: regression values of intercept ($m_{1}$) coefficients of parameters $E_{T}\left(30\right)$ and $\left(f\left(\epsilon \right)-f\left(n^{2}\right)\right)$ ($m_{2}$ and $m_{3}$ respectively) and relative importance of the two parameters (%R_ET(30)_ and %R*_f_*_(*ϵ*) − *f*(*n*2)_).

${\tilde{v}}_{CT}=m_{1}+m_{2}E_{T}\left(30\right)-m_{3}\left(f\left(\epsilon \right)-f\left(n^{2}\right)\right)$						
	$m_{1}$ (10^3^cm^−1^)	$m_{2}$ (10^3^cm^−1^·mol·kcal^−1^)	$m_{3}$(10^3^cm^−1^)	* r ^2^	%R_ET(30)_	%R*_f_*_(_*_ϵ_*_) − *f*(*n*2)_
**5a**	0.988	0.291	1.750	0.849	63	37
**5b**	1.168	0.286	1.621	0.898	59	41
**7**	1.182	0.302	1.580	0.556	11	88

* *r*^2^ corresponds to the regression coefficient of the linear fit: ${\tilde{v}}_{CT}(exp)\;vs.\;{\tilde{v}}_{CT}(cal)$ where *cal* values correspond to calculated values obtained through Equation (10) whereas *exp* values correspond to the experimentally obtained values.

## Data Availability

Data are contained within the article and Supplementary Materials.

## Supplementary Materials

The following supporting information can be downloaded at: https://www.mdpi.com/article/10.3390/nano15080598/s1. Figure S1. Ball and Stick representations of anions of **5a** and **5b** optimized using Molecular Mechanics on Avogadro 1.2.0. The UFF force field was used in both cases; Figure S2. Plot of the polarity function *f*(*ε*) of aqueous urea as a function of urea concentration; Figure S3. Plot of the polarity function *f*(*n*^2^) of aqueous urea as a function of urea concentration; Figure S4. Plot of the refractive index of aqueous urea as a function of urea concentration; Figure S5. Plot of the permittivity of aqueous urea as a function of urea concentration; Figure S6. Plot of Reichardt’s polarity scale of aqueous urea as a function of urea concentration; Figure S7. Partial FTIR spectra of **5a** (C≡N stretching band) in water and in the presence of 1 (0.5 M), 2 (1 M), and 3 (1.5 M) equivalents of urea (at a constant **5a** concentration of 0.5 M). The arrow indicates the slight blue shift in the band with increasing urea equivalents.

## Nomenclature

*a_r_*	Van der Waals cavity parameter of a solvatochromic compound
α	HBD acidity parameter (involved in KAT equation)
*a*	Coefficient of parameter α
*β*	HBA basicity parameter (involved in KAT equation)
*b*	Coefficient of parameter β
bpy	4,4′-Bipyridine
*δ*	Chemical shift (NMR)
*e*	$\mathrm{Coefficient}\;\mathrm{of}\;\mathrm{parameter}\;E_{T}^{N}$ in Equations (1) and (4) (does not correspond to the mathematical constant: Euler’s number)
*EG*	Ethylene glycol
$E_{T}^{N}$	Normalized Reichardt’s polarity scale
$E_{T}\left(30\right)$	Dimroth–Reichardt’s polarity scale
ϵ	Permittivity of the medium
$\epsilon_{0}$	Vacuum permittivity
$f\left(\epsilon \right)$	Dipolarity function
$f\left(n^{2}\right)$	Polarizability function
*FA*	Formamide
$\varphi \left(\epsilon \right)$	Dipolarity function in Suppan–Tsiamis equation
$\varphi \left(n^{2}\right)$	Polarizability function in Suppan–Tsiamis equation
$\varphi \left(\epsilon ,n^{2}\right)$	“Pure dipolarity” function: *φ*$\left(\epsilon ,n^{2}\right)$= $\varphi \left(\epsilon \right)-\varphi \left(n^{2}\right)$
HBD	Hydrogen Bond Donor
HBA	Hydrogen Bond Acceptor
*J*	Coupling constant in ^1^H-NMR
*KAT*	Kamlet–Taft–Abboud (Equation)
$k_{C}$	$Coulomb’s\;constant;\;k_{C}=\frac{1}{4\pi \varepsilon_{o}}≅8.988\cdot 10^{9}\;Nm^{2}C^{-2}\;$
LSER	Linear solvation energy relationship
$\lambda_{CT}^{5a},\lambda_{CT}^{5b}{,\lambda }_{CT}^{7}$	MLCT maximum wavelength of comound **5a**, **5b** and **7** respectively
$m_{1}$ $,m_{2},m_{3}$	Intercept and two coefficients involved in Equation (10)
$\vec{\mu_{g}}$	Ground state dipole moment
$\vec{\mu_{e}}$	Excited state dipole moment
${\tilde{v}}_{CT}^{5a}$ $,{\;\tilde{v}}_{CT}^{5b}$ $,{\;\tilde{v}}_{CT}^{7}$	MLCT maximum wavenumber of comound **5a**, **5b** and **7** respectively
${\tilde{v}}_{o}$	Intercept MLCT wavenumber involved in LSERs: 1–4
$n^{2}$	Polarizability of the medium
n	Refractive index of the medium
*NMF*	N-methyl formamide
*π**	Dipolarity/polarizability parameter involved in KAT equation
Q	Physicochemical parameter involved in the generic LSER equation:
$Q_{o}$	Intercept involved in the generic LSER equation:
*Ρ*	Density of an aqueous urea solution
*s*	Coefficient of parameter π* (KAT equation)
*TFE*	2,2,2-Trifluoroethanol
$x_{i}$	$\mathrm{Coefficient}\;\mathrm{of}\;\mathrm{parameter}\;X_{i}$
$X_{i}$	Parameter involved in the generic LSER model
