# Supplementary material for: Chromotropism of Iron(II) Complexes with Non-Symmetric Heterocyclic Ligands: Polarity Sensing in Aqueous Urea Solutions
Source: Nanomaterials (Basel). 2025 Apr 13;15(8):598. doi: 10.3390/nano15080598 (PMC12029940; doi:10.3390/nano15080598)
Supplement: Supplementary file 1 [file nanomaterials-15-00598-s001.zip › nanomaterials-3515796-supplementary.pdf]

Supplementary Data File for:

Chromotropism of Iron(II) Complexes with Non-Symmetric Heterocyclic Ligands: Polarity Sensing in Aqueous Urea Solutions

Raffaello Papadakis*

Swedish University of Agricultural Sciences, Department of Forest Biomaterials and Technology, Uppsala, 756 51, Sweden

Email: rafail.papadakis@slu.se

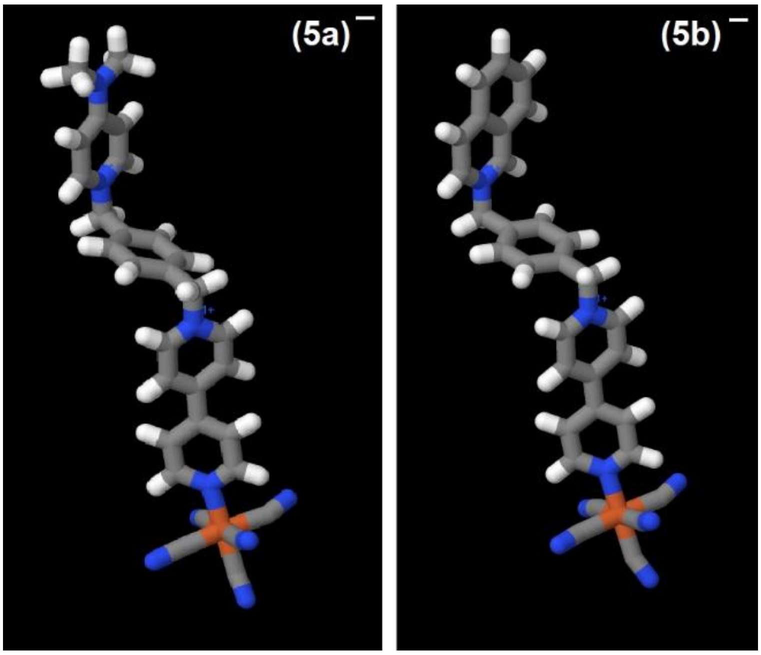

Figure S1. Ball and Stick representations of anions of 5a and 5b optimized using Molecular Mechanics on Avogadro 1.2.0. The UFF force field was used in both cases.

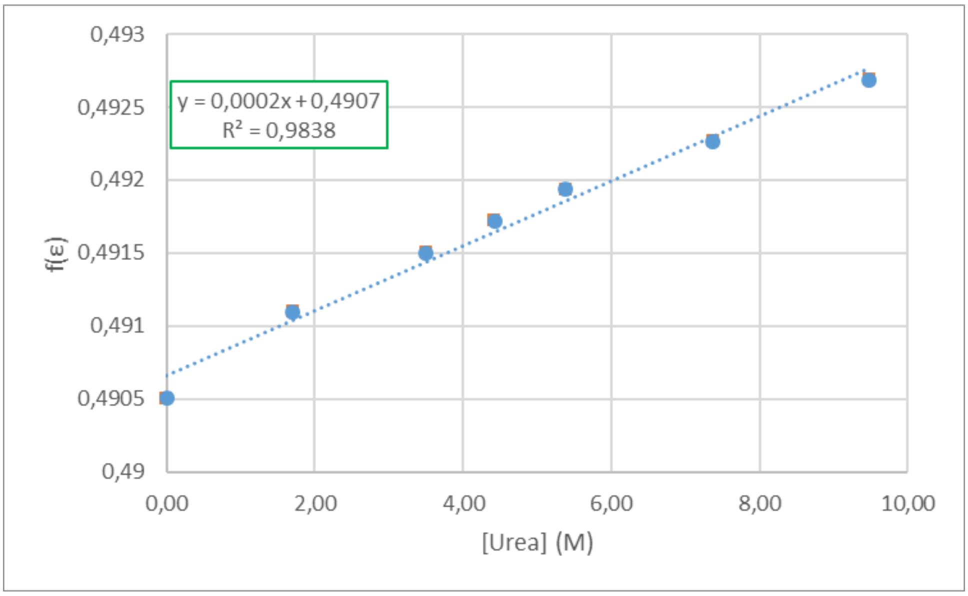

Figure S2. Plot of the polarity function *f(ε)* of aqueous urea as a function of urea concentration

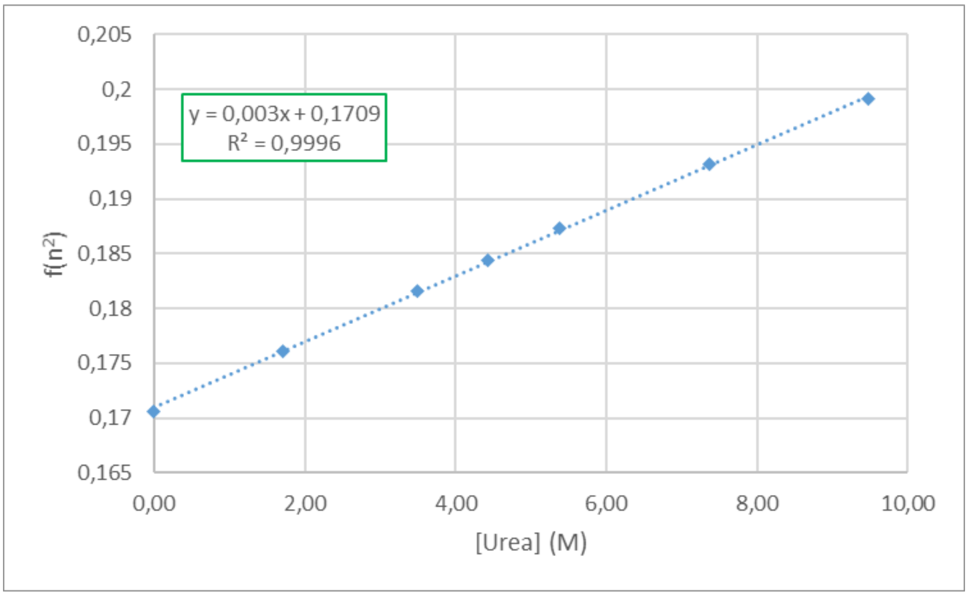

Figure S3. Plot of the polarity function $f(n^2)$ of aqueous urea as a function of urea concentration

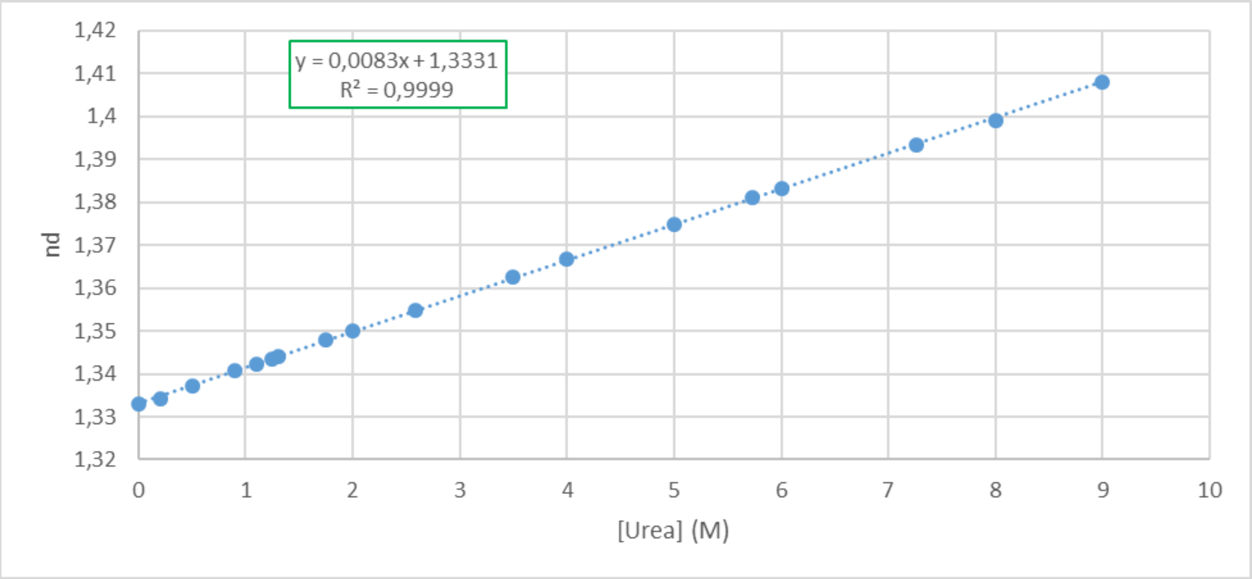

Figure S4. Plot of the refractive index of aqueous urea as a function of urea concentration

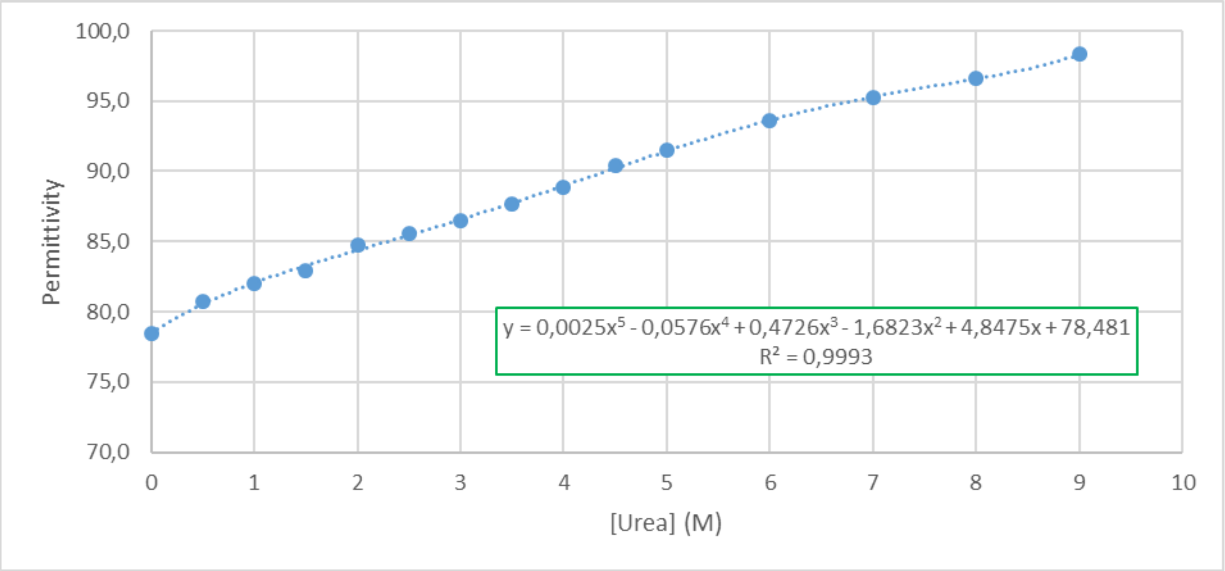

Figure S5. Plot of the permittivity of aqueous urea as a function of urea concentration

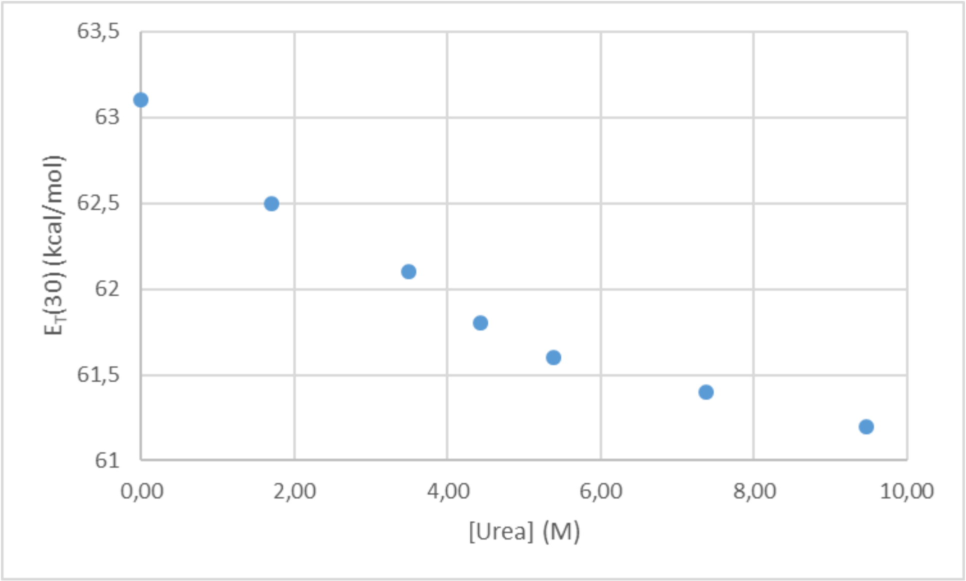

Figure S6. Plot of Reichardt's polarity scale of aqueous urea as a function of urea concentration

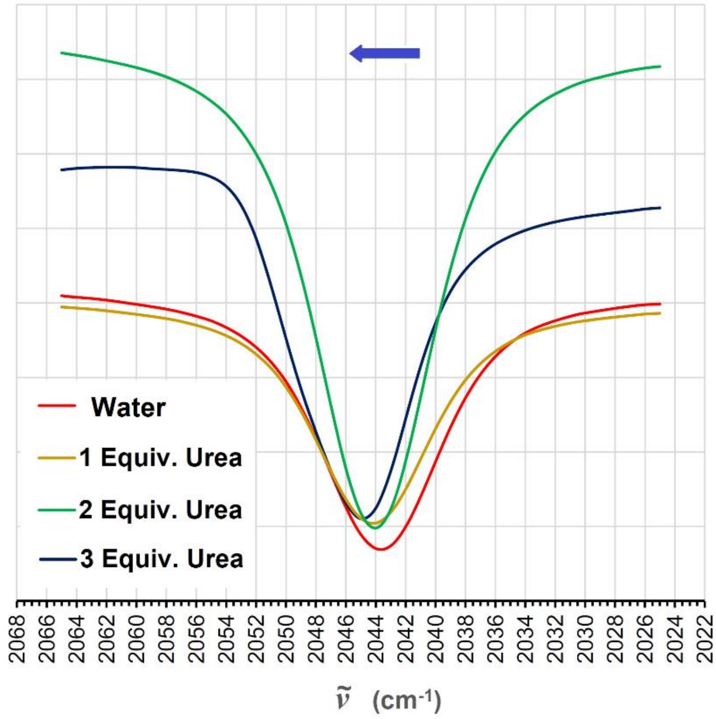

Figure S7. Partial FTIR spectra of 5a (C≡N stretching band) in water and in the presence of 1 (0.5 M), 2 (1 M), and 3 (1.5 M) equivalents of urea (at a constant 5a concentration of 0.5 M). The arrow indicates the slight blue shift in the band with increasing urea equivalents.
